# Variation in Caesarean Section Rates in the US: Outliers, Damned Outliers, and Statistics

**DOI:** 10.1371/journal.pmed.1001746

**Published:** 2014-10-21

**Authors:** Gordon C. S. Smith

**Affiliations:** 1 Department of Obstetrics and Gynaecology, University of Cambridge, Cambridge, United Kingdom; 2 NIHR Cambridge Comprehensive Biomedical Research Centre, Cambridge, United Kingdom

## Abstract

Gordon C. Smith discusses the study by Katy Kozhimannil and colleagues that examines variations in cesarean section rates in the US and argues for the need for high-quality routine data collection to better understand the reasons for these variations.

*Please see later in the article for the Editors' Summary*

**Linked Research Article**

This Perspective discusses the following new study published in *PLOS Medicine*:

Kozhimannil KB, Arcaya MC, Subramanian SV (2014) Maternal Clinical Diagnoses and Hospital Variation in the Risk of Cesarean Delivery: Analyses of a National US Hospital Discharge Database. PLoS Med 11(10): e1001745. doi:10.1371/journal.pmed.1001745

Katy Kozhimannil and colleagues use a national database to examine the extent to which variability in cesarean section rates across the US in 2009–2010 was attributable to individual women’s clinical diagnoses.

Prior to the 20th century, birth could only safely be achieved by vaginal delivery of the fetus. This natural process was accompanied by high mortality rates for both mothers and infants. The development of safe abdominal delivery was one of a series of interventions that led to dramatic falls in maternal and perinatal mortality in high-income countries in the 20th century. Today, lack of access to safe caesarean delivery is a major global public health problem, and provision of the procedure in low-income settings is a key element of programmes aimed at reducing the substantial proportion of the global burden of death and disability that follows pregnancy complications [1].

However, in many higher income countries around the world, caesarean section evolved during the course of the 20th century from being a rarely performed, desperate measure, to being a life-saving occasional intervention, to being a core component of safe obstetric care, ultimately to become the most commonly performed laparotomy and a major focus of concerns around unnecessary medical intervention and avoidable healthcare costs. Women and doctors grapple with the concept of clinically indicated and non-clinically indicated procedures. However, in most cases, there is no absolute indication. The decision to perform a caesarean section involves balancing multiple risks: short- and long-term, maternal and foetal, for and against performing the procedure [2]. Judging the balance of these risks for an individual woman in many ways requires more skill than performing the procedure. Moreover, many women approach the decision with firmly held prior beliefs that, quite naturally, are not wholly based on an objective balancing of the probabilities of adverse events.

How does this decisional complexity manifest itself in the real world? This question is addressed by the research article of Katy Kozhimannil and colleagues published in this week’s *PLOS Medicine* [3]. The authors show that between-institution differences in the rates of caesarean section in the United States were not explainable purely by random variation. In an analysis of almost 1.5 million births in a representative sample of 20% of obstetric units in the US, they demonstrated an excess of outliers, i.e., units with unexpectedly low or high rates of caesarean section. If variation had simply been due to the play of chance, they would have expected 70 outliers. What they observed were 541 outliers, i.e., an almost 8-fold excess. The variability was not affected by adjustment for a range of demographic and obstetric characteristics that were recorded in the dataset employed.

Major determinants of the prior risk of caesarean section include nulliparity, induction of labour (primarily through the indication rather than the procedure itself) [4], previous caesarean delivery, multiple pregnancy, malpresentation, and prematurity [5]. The importance of these factors has led to analysis of variation in caesarean section rates being performed within groups, such as "nulliparous, single cephalic, ≥37 weeks, in spontaneous labour". Analysis by group allows assessment of whether an overall high (or low) caesarean section rate within an institution is observed across a range of clinical scenarios in which the decision-making processes may differ. A consistently high rate of caesarean section across diverse categories may indicate bias towards performing the procedure in the absence of a strong clinical indication. Conversely, an institution may have a high overall rate due to an excess of one or more groups that have inherently higher rates of caesarean birth. Such analyses then allow clinicians to identify interventions which are most likely to reduce the number of unnecessary surgical births, such as improved utilisation or performance of external cephalic version for term breech presentation, or facilitating better uptake of vaginal birth after caesarean section when appropriate. The paper of Kozhimannil et al. lacked the basic information available that is required to analyse rates by subgroups. This limits our ability to understand from this analysis why the rates varied so much and how the variation might be addressed.

A series of further questions remains. First, even within the categories of caesarean section, there are important maternal characteristics, unmeasured in the present study, that can influence the risk, such as height, body mass index, and post-dates pregnancy [6],[7]. The lack of information on these factors adds further to concerns that the persistence of an excess of outliers in the analyses by Kozhimannil and colleagues may simply reflect the influence of unmeasured maternal or obstetric characteristics. Second, if the high level of variation observed is due to institutional factors, such as maternal or physician preference in the presence of ambiguous or marginal indications, why was the variation greater for high-risk situations, where practice and maternal choices might have been expected to be more consistent? Third, what was the relationship between the observed and expected proportion of caesarean section and the proportion of adverse maternal and perinatal outcomes? Did the units with lower proportions of caesarean section achieve the lower rate at the expense of higher rates of serious adverse events? Did units with high rates of caesarean delivery also have higher rates of serious maternal morbidity? Finally, given that between 1 and 1.5 million women are delivered by caesarean section each year in the US [8], and given the associated costs of these procedures (financial and clinical), how can it be that we can only guess at the effects of parity and gestational age on variation in caesarean section rates in the world's richest nation? The weaknesses in routine collection of maternity data in the US are well recognised [9]. Why is the collection of high quality maternity data such a low priority?

As researchers, we need to continue to strive to understand the determinants and consequences of varying rates of caesarean section and to identify appropriate responses. But this can only be done if providers recognise that the collection of high quality routine data—and making it available for the purposes of research—is an essential element of safe health care.
